# Reliability and validity of a graphical computerized adaptive test Longshi scale for rapid assessment of activities of daily living in stroke survivors

**DOI:** 10.1038/s41598-024-57671-1

**Published:** 2024-04-01

**Authors:** Jing Zhou, Fubing Zha, Fang Liu, Li Wan, Mingchao Zhou, Jianjun Long, Miaoling Chen, Kaiwen Xue, Yulong Wang

**Affiliations:** 1 Department of Rehabilitation, First Affiliated Hospital of Shenzhen University/Shenzhen Second People’s Hospital, 3002 Sungang West Road, Futian District, Shenzhen, 518035 Guangdong China; 2 Department of Neurology, Institutes of Brain Science, State Key Laboratory of Medical Neurobiology and MOE Frontiers Center for Brain Science, Institute of Biological Science, Zhongshan Hospital, Fudan University, Shanghai, 200032 China

**Keywords:** Activities of daily living, Barthel index, Computer adaptive test-Longshi Scale, Stroke, Computational biology and bioinformatics, Health occupations, Medical research

## Abstract

Stroke survivors frequently experience difficulties in daily activities, such as bathing, feeding, and mobility. This study aimed to evaluate the reliability and validity of a computer-adaptive test-Longshi scale (CAT-LS) for assessing activities of daily living (ADL) in stroke survivors. This cross-sectional study collected data using an electronic application. The ADL function of stroke survivors in rehabilitation departments of hospitals was assessed using both the CAT-LS and BI. Correlations between the CAT-LS and Barthel index (BI) and concurrent validity were evaluated using Pearson’s correlation test and multiple linear regression. Interrater reliability was evaluated using the intraclass correlation coefficient based on a two-way random effect. The internal consistency of the CAT-LS was assessed using Cronbach’s coefficient (α) and corrected item-total correlations. Overall, 103 medical institutions in China were used in this study. In total, 7151 patients with stroke were included in this study. The CAT-LS classified patients into three ADL groups (bedridden, domestic, and community) with significantly different BI scores (P < 0.05). The CAT-LS results obtained using the decision-tree scoring model were consistent with the scores for each BI item. A strong correlation was observed between CAT-LS and BI (Pearson’s r: 0.6–0.894, P < 0.001). The CAT-LS demonstrated good internal consistency (Cronbach’s α, 0.803–0.894) and interrater reliability (ICC, 0.928–0.979). CAT-LS is time-efficient and requires < 1 min to administer. The CAT-LS is a reliable and valid tool for assessing ADL function in stroke survivors and can provide rapid and accurate assessments that reduce the burden on healthcare professionals. Further validation of this tool in other populations and settings is necessary.

Study registration number: No.: ChiCTR2000034067; http://www.chictr.org.cn/showproj.aspx?proj=54770.

## Introduction

Stroke survivors frequently experience difficulties in daily activities and quality of life^1^. A significant proportion of stroke survivors, ranging from 36 to 89%, experience one or more functional disabilities^2^. Therefore, assessing activities of daily living (ADL) is crucial for clinicians in determining treatment methods and enhancing independence^3^. A short, reliable, and valid ADL measure is required to be clinically useful and reduce the burden on clinicians and patients^4^.

ADL includes basic ADL (BADL) and instrumental ADL (IADL)^5^. BADL measures, including the Barthel index (BI) and Functional Independence Measure, tend to have ceiling effects, whereas IADL measures tend to have floor effects^6^. The BI is a measure of public-domain ADL that is commonly used in clinical trials to assess patients with stroke^7^. The tool assesses the following 10 activities related to BADLs: bowel management, bladder control, grooming, feeding, toilet use, transferring, movement, dressing, climbing stairs, and bathing^8^. These activities are fundamental to living in a social world. Many IADL scales are closely related to environmental performance^9^. IADL scales comprised varying numbers of items covering the domains of housework, work/leisure, outdoor activities, dressing outside, shopping, and eating with guests^9^. However, simultaneously assessing all ADLs and IADLs may require more time, and would be physically demanding for both the patient and the clinician. Some patients do not need to be evaluated for all activities. For example, some long-term bedridden patients are completely unable to walk or climb stairs and do not need to be asked about this item by the assessor.

Therefore, to address this problem, our previous study developed the Longshi scale (LS), which is a graphical tool for assessing ADL based on the International Classification of Functioning, Disability, and Health (ICF) guidelines^10,11^. The scale categorizes patients into the following three groups based on ADL capability: bedridden, domestic, and community^12^. Each group includes 3 different items, which provides a practical solution for reducing the ADL scale length by linking ADL assessment with the scope of activities. The LS has demonstrated high interrater reliability (0.877–0.955) and test–retest reliability (0.921–0.984)^11^. Additionally, using smartphone video technology in LS for assessing ADL in stroke survivors can be effectively implemented in remote clinical settings^13^.

To streamline the LS evaluation process, we integrated computerized adaptive testing (CAT) with the inner logic of the programming in LS(CAT-LS)^14,15^. Computerized adaptive testing (CAT) is a proven method for the efficient, reliable, and valid assessment of health-related functions^16,17^. CAT leverages item response theory to dynamically select the most relevant questions based on prior responses, resulting in more concise questionnaires with enhanced precision^18^, and reducing the administrative burden on patients^19,20^, which is similar to the LS-categorizing assessment rule.

Nevertheless, the validation and interrater reliability of CAT-LS in hospitalized stroke survivors remain unestablished. Consequently, the study aims to evaluate CAT-LS concurrent validity, internal consistency, and inter-rater reliability in stroke survivors. Building upon the robust psychometric characteristics demonstrated by conventional LS assessments^11,13,15,21^, we postulated that CAT-LS would emerge as a reliable and valid instrument for assessing ADL in our target population.

## Methods

### Study design and participants

This multicenter cross-sectional study was conducted in 103 hospitals in 23 cities in China from September 2018 to August 2019 and involved 7151 cerebral stroke survivors using cluster sampling. The specific inclusion criteria were as follows: (1) individuals aged between 18 and 90 years and (2) those diagnosed with a stroke. The type of stroke was determined based on the initial diagnosis from medical history, according to the 10th revision of the International Classification of Diseases^22^. The exclusion criteria were as follows: diagnosis of subarachnoid hemorrhage.

### CAT-LS development procedure

The CAT-LS development procedure comprises three phases. In phase 1, a total of 11 items from the BI, IADL, and ICF were selected to comprise the traditional paper version of the LS (including bladder and bowel management, feeding, entertainment, toileting, grooming, and bathing, cooking, community mobility, shopping, social interaction, transfer out of bed and return, and transfer out of the door and return) (Table 1)^23,24^. Out of the total 11 items, seven originated from BI while the remaining four were derived from ICF and IADL. Over 80% of the ICF items primarily focused on “activities”, which encompass “individuals performing specific tasks or actions”, as well as “physical functions”^24^. It is crucial not to overlook the social participation requirements of patients^24^. Selection of the 4 social participation entries in ICFs and IADLs that are of most concern to patients based on a previous study (Table 1)^11^. In phase 2, a CAT system based on the LS decision tree was presented, and validation datasets were collected using a smart mobile application named ‘Quicker Recovery Line (QRL)’^23,25^. In phase 3, concurrent validity and inter-rater reliability of CAT-LS and the correlation with BI in stroke survivors were calculated.

**Table 1:** Comparison of the proposed CAT-LS, BI, and ICF.

Items	BI item	IADL	CAT-LS	ICF
1	Feeding	Eating with guests	F1b feeding	b525 defecation functions\d550 eating
2	Bathing		F2b grooming and bathing	d510 washing oneself
3	Grooming		F2b grooming and bathing	d510 washing oneself
				d520 caring for body part
4	Dressing			
5	Bowel management		F1a bladder and bowel management	
6	Bladder management		F1a bladder and bowel management	b620 urination functions
7	Toileting		F2a toileting	d530 toileting\d5308 Toileting, other specified
8	Bed/chair transfer		Q1: able to transfer out of bed and return	d410 changing basic body position
9	Ambulation	Outdoor activities	F3a community mobility	d450 walking
10	Stair climbing			
11		Outdoor activities	Q2: able to transfer out of the door and return	d4602 moving around outside the home and other building
12		Leisure	F1c entertainment	d3600 using telecommunication devices
13		Meal preparation	F2c cooking	d640 doing housework\d630 preparing meals
14		Shopping	F3b shopping	d6200 Shopping
15		Transportation	F3c social interaction	d470 Using transportation

Subscales: motor = items 1–11; and social participation = 12–15.

### Data collection

The assessment was conducted using smart mobile devices (mobile phones or tablets). Before the formal assessment, the investigators explained the study contents to all participants.

All data were recorded and uploaded on the ‘QRL’. First, one healthcare professional logged into the QRL account and created electronic forms online. The demographic information of all participants, including basic information and health status, was recorded. Second, patients’ BI and CAT-LS scores were on a face-to-face interview basis and were collected by interviewing stroke survivors, caregivers, and doctors^21,25^. Once the data collection was completed, it could not be changed. The data were from our previous study^23^. Missing data were handled by conducting reinterviews.

### ADL evaluation

We assessed Activities of Daily Living (ADL) using two methods: the CAT-LS and the BI scale^26,27^. For the first day’s assessment, the choice of whether to use the CAT-LS or the BI scale was made randomly each time. The performance of the CAT-LS and BI scales was assessed by medical professionals and therapists respectively. To check the consistency in evaluations between different raters, the same assessors evaluated the ADL of stroke survivors once more on the second day, measuring the interrater reliability of the CAT-LS. These assessments were carried out one after the other during a single clinical visit, and we recorded the time taken for each assessment in seconds.

The BI was designed in traditional electronic format with the same specifications as the paper version. Physicians and therapists can submit assessment results only after all items are selected. BI consists of 10 ADL tasks, including feeding, bathing, grooming, dressing, bowel management, bladder management, toileting, bed/chair transfer, wheelchair, climbing stairs, and range based on the level of physical assistance required to complete the task. These are based on the level of physical assistance required to complete the task^28^. Most of the items were scored in a range of 0–10, where scores of 0, 5, and 10 indicated an inability to perform the task, need for assistance, and ability to perform the task independently, respectively, for a combined total of 100 points. Bathing, wheelchair, and grooming items were scored in a range of 0–5, where scores of 0 and 5 indicated inability to perform the task and full ability to perform the task independently, respectively. In addition, the Bed\chair transfer items were scored on a scale ranging from 0 to 15, where a score of 5–10 indicated the need for assistance in completing the task, and a score of 15 indicated a full ability to complete the task independently (Table 2).

**Table 2:** Barthel index scores and the capability to perform ADL.

Items	Unable to perform the task	Needs assistance	Fully independent
Feeding	0	5	10
Bathing	0	0	5
Grooming	0	0	5
Dressing	0	5	10
Bowel management	0	5	10
Bladder management	0	5	10
Toileting	0	5	10
Bed/chair transfer	0	5–10	15
Wheelchair	0	0	5
Climbing stairs	0	5	10
Range	0		100

**Figure 1:**
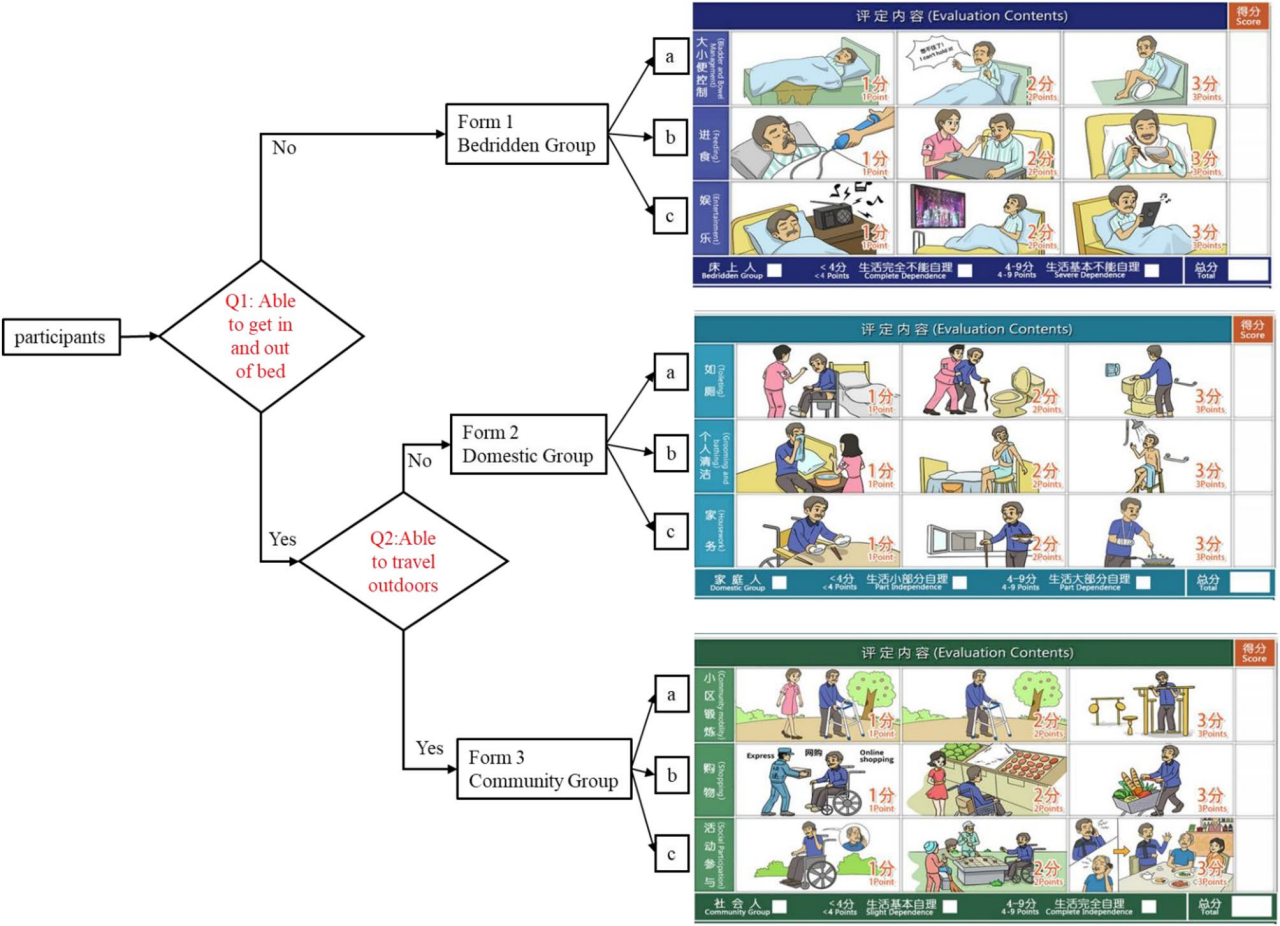
Process for CAT-LS assessment.

The CAT-LS used item response theory. Item response theory is the statistical basis for testing the fit of the data model to estimate the difficulty of the questions and the respondent’s ability^29^. The CAT-LS decision tree functions as a flowchart, commencing with a primary question—“Can you get off the bed?”, and subsequently diverging into different branches based on the outcomes of the initial decision. Further branching occurs based on the results of a subsequent question—“Can you go outside?”, enabling a visual representation of the outcomes of a comprehensive ADL assessment. This approach facilitates a rapid comprehension of a patient’s ADLs. Participants were asked about their ability to get in and out of bed to begin the assessment^30^. If they answered “No”, they are categorized in the bedridden group and only the items in Form 1 are displayed on the application interface, including bladder and bowel management, feeding, and entertainment (Fig. 1). If they answered ‘YES’, they were asked a second question regarding their ability to travel outside their house and return. If they answered ‘NO’, they were categorized into the domestic group, and only items in Form 2 were shown, including toileting, personal cleaning, and housework. If they answered ‘YES’, they were categorized into a community group, and only items in Form 3 were evaluated, including community mobility, shopping, and social participation (Fig. 1). Subsequently, individuals only needed to be evaluated on items corresponding to their functional level to accurately estimate the functional level and improve evaluation efficiency.

After categorization, the patients in each group were evaluated using a 3-point Likert scale as follows: (1) bedridden (including bladder and bowel management, feeding, and entertainment), (2) domestic (including toileting, grooming, and housework), and (3) community (including community mobility, shopping, and social participation) groups. Each item corresponded to three multiple-choice questions with different abilities, and all alternatives were presented as a situation map (Fig. 1). Each item was scored as follows: 1 for maximum or complete dependence, 2 for partial independence, and 3 for maximum or complete independence. The evaluation was completed once the three items on the subscale were selected. The total scores of each group were 3–9 (Fig. 1)^30^, and accordingly, the CAT-LS was categorized into six grades, and subsequently, the CAT procedure ended (Table 3).

**Table 3:** Scoring guidelines for the ICF qualifiers.

From	CAT-LS score	CAT-LS grade	Description
From 1	3 points	1 = Complete Dependence	The person does not perform the activity at all
From 1	4–9 points	2 = Severe Dependence	The activity is carried out completely dependently; continuous help (guiding, support, or effective help) from others is needed. The person experiences severe problems in performance
From 2	3 points	3 = Part Independence	The person can perform the activity in a very limited range
From 2	4–9 points	4 = Part Dependence	The activity is carried out independently but sometimes help is needed. There are moderate limitations in performance; the person is less result oriented and less adequate. There are faults in performance
From 3	3 points	5 = Slight Dependence	The activity is carried out completely independently; no help from others is needed but mild limitations are present: less frequent use of, the more simplified form of the activity (e.g. only a few functions of technological equipment). The person needs more time, is slower, less energetic, and has difficulties learning something new. The person is less flexible, inventive, creative, and more rigid
From 3	4–9 points	6 = Complete Independence	The activity is carried out completely independently; no help from others is needed. There are no limitations, the person carries out the activity at a normal frequency and is adequate, flexible, inventive, and creative (e.g. the person can use all functions of technological equipment)

### Automatic quality control process

In this multicenter study, evaluators obtained the electronic data of CAT-LS and BI from smartphone device terminals located in different centers and were transmitted to the cloud server through the application named ‘QRL’^25^. Data quality was evaluated using the built-in automatic quality control system of the cloud server. For each day, if the data quality for the same evaluator was compromised, all evaluation data of the evaluator on that day were discarded. The built-in logic of quality control is presented in Table 4.

**Table 4:** Inner logic for automatic quality control.

Quality control rules
1. In CAT-LS rated as Bedridden, but in BI the bed chair transfer gain 15 points
2. In CAT-LS is rated as Domestic, but in BI the bed chair transfer gain 0 points, or the total BI score is 0
3. In CAT-LS rated as Community, but in BI the bed chair transfer gained 0 points, or the total BI score is 0
4. For Bedridden: 1 point for CAT-LS defecation control, but 10 points for BI defecation control
5. For Bedridden: 3 points for CAT-LS defecation control, but 0 points for BI control
6. For Bedridden: 1 point for CAT-LS feeding, but 10 points for BI feeding
7. For Bedridden: 3 points for CAT-LS feeding, but 0 points for BI feeding
8. For Domestic: 1 point for CAT-LS self-cleaning, but 5 points for BI bathing or decoration
9. For Domestic: 1 point for CAT-LS toileting, but 10 points for BI toileting
10. For Domestic: 3 points for CAT-LS toileting, but 0 points for BI toileting
11. Personal information indicated living alone but defecation or feeding in CAT-LS and BI were low

### Statistical analysis

Statistical analyses were performed using SPSS Statistics 25. The Kruskal–Wallis test, t-test, and chi-square test were performed to analyse mean differences between LS groups (i.e., bedridden, domestic, and community groups). Differences among CAT-LS groups were evaluated by comparing the means and standard deviations of the different scores of the original BI measures.

### Internal consistency and concurrent validity

The CAT-LS was trained to predict the total BI scores using the raw scores of each item on the CAT-LS. We utilized generalized linear models with total BI as the dependent variable and each item of LS as the independent variable. The internal consistency of the CAT-LS was assessed using Cronbach’s coefficient (α) and corrected item-total correlations. Pearson’s correlation test and multiple linear regression analysis were used to analyse the concurrent validity of the CAT-LS. Notably, concurrent validity was considered strong if Pearson’s correlation coefficient was ≥ 0.75^31^.

### Interrater reliability

The interrater reliability of the CAT-LS was evaluated using the kappa coefficient (κ) and intraclass correlation coefficient based on a two-way random effect (ICC2,1)^32,33^. ICC values were categorized as ‘poor’ (ICC < 0.5), ‘moderate’ (0.5–0.75), ‘good’ (0.75–0.9), and ‘excellent’ (ICC > 0.9)^33^. The kappa values were defined as ‘poor’ (κ < 0.20), ‘fair’ (0.21–40), ‘moderate’ (0.41–60), ‘good’ (0.61–80), and ‘very good’ (κ = 0.81–1.00) agreement^32^.

### Suppliers

A smart mobile application named “Quicker Recovery Line (QRL)”.

### Ethics approval and consent to participate

This study protocol was approved by the Medical Ethics Committees of Shenzhen Second People’s Hospital. The study was registered in the Chinese Clinical Trial Registry (No.: ChiCTR2000034067) on June 22, 2020. All inpatients or their proxies were invited to participate in this study after obtaining informed consent before collecting their information. All authors confirmed that all methods were carried out following the research protocol approved by the ethics committee.

## Results

### Population characteristics

Table 5 presents the baseline demographic characteristics of 7151 stroke survivors. The CAT-LS classified 4020 (56.2%), 2050 (28.7%), and 1081 (15.1%) patients into the bedridden, domestic, and community categories, respectively. The mean age of the participants was 67.6 ± 15.0 years, and a statistically significant difference was found in the mean age among the three groups. Hypertension was the most common comorbidity in the community group, affecting 672 (62.16%) participants, followed by diabetes mellitus (255; 23.63%), hyperlipidemia (116; 10.75%), heart disease (172; 15.93%), and kidney disease (25; 2.31%).

**Table 5:** Demographic information on stroke survivors.

Characteristics		Total		Functional status						Kruskal–Wallis/χ^2^	P value
		n/mean	%/SD	Bedridden group		Domestic group		Community Group			
				n/mean	%/SD	n/mean	%/SD	n/mean	%/SD		
Total		7151	100.0	4020	56.2	2050	28.7	1081	15.1		
Type of stroke	Hemorrhagic	2012	28.1	1270	31.6	550	26.8	192	17.8	83.0	< 0.001
	Ischemic	5139	71.9	2750	68.4	1500	73.2	889	82.2		
Age (year)		67.6	15	70.1	14.7	65.2	14.8	62.2	14.2		< 0.001
Duration (month)		2.5	4.4	2.6	4.5	2.4	4.1	2.3	4.5		0.068*
											0.076^†^
											1^#^
Gender	Male	4303	60.2	2270	56.5	1327	64.7	706	65.4	52.6	< 0.001
	Female	2845	39.8	1748	43.5	723	35.3	374	34.6		
Smoke	No	5398	76.1	3176	79.6	1447	71.3	775	72.4	60.8	< 0.001
	Yes	1694	23.9	815	20.4	584	28.8	295	27.6		
Alcohol	No	6396	90.1	3681	92.2	1788	87.8	927	86.3	48.9	< 0.001
	Yes	706	9.9	311	7.8	248	12.2	147	13.7		
Hypertension	No	1921	26.9	961	23.9	551	26.9	409	37.8	83.7	< 0.001
	Yes	5223	73.1	3054	76.1	1497	73.1	672	62.2		
Diabetes mellitus	No	5152	72.3	2856	71.3	1472	72.1	824	76.4	11.1	0.004
	Yes	1976	27.7	1151	28.7	570	27.9	255	23.6		
Hyperlipidemia	No	6427	90.2	3667	91.5	1797	88.1	963	89.3	19.9	< 0.001
	Yes	699	9.8	339	8.5	244	12.0	116	10.8		
Heart disease	No	5445	76.2	2894	72.0	1643	80.3	908	84.1	93.9	< 0.001
	Yes	1699	23.8	1123	28.0	404	19.7	172	15.9		
Kidney disease	No	6835	95.8	3830	95.5	1950	95.5	1055	97.7	10.9	0.004
	Yes	297	4.2	181	4.5	91	4.5	25	2.3		

*Comparison between bedridden and domestic groups.

^†^Comparison between domestic and community groups.

^#^Comparison between bedridden and community groups.

### Classification and regression tree of ADL assessment using CAT-LS

**Figure 2:**
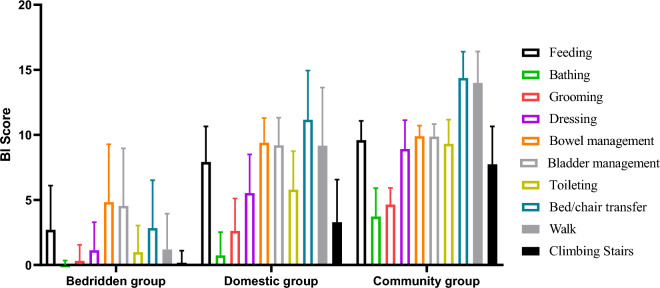
BI scores of each item among the three CAT-LS groups.

Table 6 displays the BI scores corresponding to the following three groups: bedridden (18.7 ± 18.8), domestic (64.8 ± 18.9), and community (93.5 ± 12.8). Patients in the bedridden group had lower scores than those in the domestic group in the following BI categories: bathing (0.0 ± 0.3), grooming (0.3 ± 1.2), dressing (1.1 ± 2.2), toileting (1.0 ± 2.0), bed/chair transfer (2.8 ± 3.7), walking (1.2 ± 2.8), and climbing stairs (0.2 ± 0.9). In the community group, BI scores for feeding (9.6 ± 1.5), grooming (4.6 ± 1.3), dressing (8.9 ± 2.2), bowel management (9.9 ± 0.8), bladder management (9.9 ± 1.0), toileting (9.3 ± 1.7), bed/chair transfer (14.4 ± 2.0), and walking (14.0 ± 2.4) tended towards the highest scores of 10/15 (Table 6, Fig. 2). Notably, the bed/chair transfer and walking categories had almost perfect scores in the community group. The CAT-LS results based on the decision-tree scoring model were consistent with the scores for each BI item. However, the median scores for 10 BI items significantly differed among the three CAT-LS groups (Table 7).

**Table 6:** BI scores in three CAT-LS groups.

Total BI/items	Bedridden group (N = 4020)				Domestic group (N = 2050)				Community group (N = 1081)				P-value
	Mean	SD	95% CI		Mean	SD	95% CI		Mean	SD	95% CI		
			Lower bound	Upper bound			Lower bound	Upper bound			Lower bound	Upper bound	
Total BI	18.7	18.8	18.2	19.3	64.8	18.9	64.0	65.6	92.1	12.5	91.3	92.8	< 0.001
Feeding	2.7	3.4	2.6	2.8	7.9	2.7	7.8	8.0	9.6	1.5	9.5	9.7	< 0.001
Bathing	0.0	0.3	0.0	0.0	0.7	1.8	0.7	0.8	3.7	2.2	3.6	3.9	< 0.001
Grooming	0.3	1.2	0.3	0.4	2.6	2.5	2.5	2.7	4.6	1.3	4.6	4.7	< 0.001
Dressing	1.1	2.2	1.1	1.2	5.5	3.0	5.4	5.7	8.9	2.2	8.8	9.1	< 0.001
Bowel management	4.8	4.5	4.7	5.0	9.4	1.9	9.3	9.5	9.9	0.8	9.9	10.0	< 0.001
Bladder management	4.5	4.4	4.4	4.7	9.2	2.1	9.1	9.3	9.9	1.0	9.8	9.9	< 0.001
Toileting	1.0	2.0	0.9	1.1	5.8	3.0	5.7	5.9	9.3	1.9	9.2	9.4	< 0.001
Bed/chair transfer	2.8	3.7	2.7	3.0	11.2	3.8	11.0	11.3	14.4	2.0	14.3	14.5	< 0.001
Walk	1.2	2.8	1.1	1.3	9.2	4.5	9.0	9.4	14.0	2.4	13.9	14.1	< 0.001
Climbing stairs	0.2	0.9	0.1	0.2	3.3	3.3	3.2	3.4	7.7	2.9	7.6	7.9	< 0.001

**Table 7:** Comparisons of BI-item scores among three CAT-LS groups.

Comparisons (median difference)	Total BI	Feeding	Bathing	Grooming	Dressing	Bowel management	Bladder management	Toileting	Bed/chair transfer	Walk	Climbing stairs
Bedridden group vs domestic group	47.3*	5.3*	1.0*	2.5*	4.7*	4.5*	4.6*	4.9*	8.5*	8.0*	3.3*
Bedridden group vs community group	75.1*	6.8*	4.3*	4.3*	8.1*	5.1*	5.4*	8.5*	11.7*	12.8*	8.1*
Domestic group vs community group	27.8*	1.5*	3.3*	1.8*	3.4*	0.6*	0.8*	3.6*	3.2*	4.8*	4.8*

*Correlation is significant at the 0.05 level (2-tailed).

### CAT-LS results (CAT-LS grades and LS scores) had a strong correlation with BI scores

Table 8 displays the results of the correlation analysis between the CAT-LS and BI scores of stroke survivors. A strong correlation was observed between the CAT-LS and BI scores (Pearson’s r = 0.894, P < 0.0001), indicating that the CAT-LS grades and item scores were good indicators of the functional status of stroke survivors. Pearson’s r values ranged from 0.529 to 0.799 (P < 0.001) between CAT-LS items and BI total scores and from 0.600 to 0.856 (P < 0.001) between CAT-LS grades and BI items.

**Table 8:** Correlation of CAT-LS scores with BI total score and BI item scores.

CAT-LS items	Total BI	BI item score									
		Feeding	Bathing	Grooming	Dressing	Bowel management	Bladder management	Toileting	Bed/chair transfer	Walk	Stairs
F1a. Bladder and bowel management	0.799**	0.591**	0.050**	0.284**	0.433**	0.839**	0.846**	0.448**	0.612**	0.341**	0.163**
F1b. Feeding	0.752**	0.769**	0.081**	0.331**	0.471**	0.639**	0.635**	0.451**	0.597**	0.419**	0.186**
F1c. Entertainment	0.700**	0.615**	0.070**	0.264**	0.448**	0.645**	0.643**	0.404**	0.570**	0.353**	0.158**
F2a. Toileting	0.738**	0.352**	0.370**	0.431**	0.492**	0.176**	0.182**	0.695**	0.578**	0.657**	0.578**
F2b. Grooming and bathing	0.662**	0.333**	0.577**	0.486**	0.523**	0.140**	0.163**	0.533**	0.468**	0.472**	0.529**
F2c. Housework	0.529**	0.229**	0.439**	0.344**	0.454**	0.098**	0.108**	0.454**	0.348**	0.396**	0.469**
F3a. Community mobility	0.649**	0.300**	0.411**	0.354**	0.491**	0.143**	0.141**	0.475**	0.501**	0.588**	0.566**
F3b. Shopping	0.650**	0.316**	0.474**	0.337**	0.508**	0.191**	0.186**	0.458**	0.450**	0.530**	0.573**
F3c. Social participation	0.602**	0.315**	0.451**	0.350**	0.493**	0.154**	0.160**	0.425**	0.414**	0.475**	0.503**
CAT-LS grade	0.894**	0.747**	0.600**	0.697**	0.799**	0.635**	0.643**	0.829**	0.841**	0.856**	0.761**

**Correlation is significant at the 0.01 level (2-tailed).

**Figure 3:**
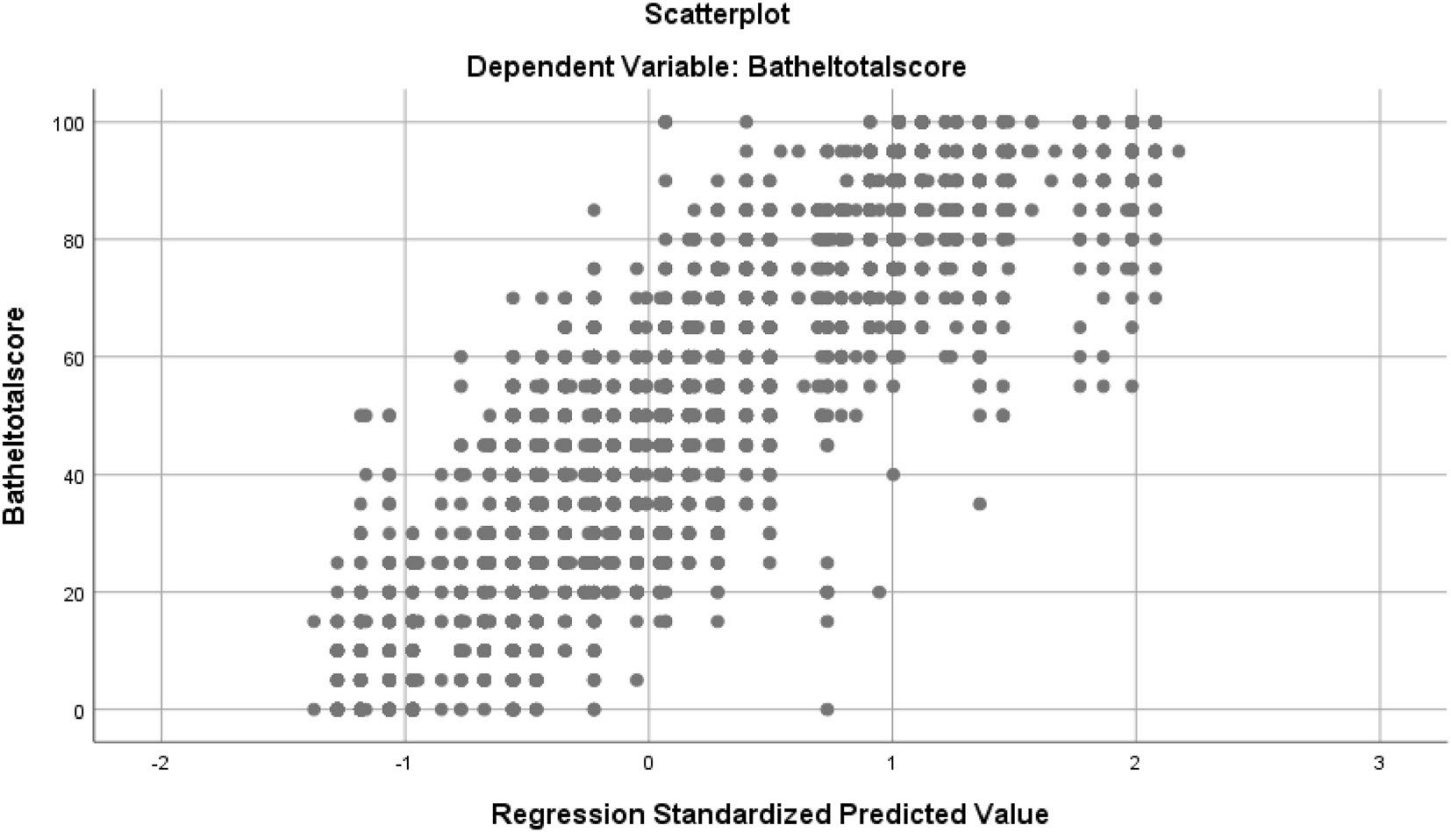
Scatter diagram illustrating the CAT-LS results (CAT-LS grades and LS scores) and BI total scores.

A scatter plot was generated to illustrate the relationship between the CAT-LS and BI scores (Fig. 3), showing the linear fitting results. The plot indicated a positive correlation between CAT-LS results and BI total scores. Moreover, a linear relationship was found between the LS results and BI total scores, and the coefficient of determination (R^2^) was 0.874. High R^2^ values indicated that the CAT-LS results were closely associated with the model’s predictions of the BI total score. To determine the correlation between the BI scale and CAT-LS scale scores, we propose the formula: BI total score =  − 44.9 + 30.44 × LS Grade + 16.14 × (F1a/F2a/F3a) + 6.79 × (F1b/F2b/F3b) − 3.04 × (F1c/F2c/F3c) (R^2^ = 0.874). F1a represents the score of the bladder and bowel item, F1b represents the score of the feeding item, F1c represents the score of the entertainment item, F2a represents the score of the toileting item, F2b represents the score of the grooming and bathing item, F2c represents the score of the housework item, F3a represents the score of the exercise in the community mobility item, and F3b represents the score of the shopping item. F3c represents the social participation score.

### Floor and ceiling effects, internal consistency, and interrater reliability of the CAT-LS

Table 9 presents the floor and ceiling effects, internal consistency, interrater reliability, and concurrent validity of the CAT-LS. The floor and ceiling effects of CAT-LS grade were 19.2%/11.7%, respectively. Internal consistency, as measured using Cronbach’s α, was high for all three subscales (bedridden, 0.847; domestic, 0.723; and community, 0.868). The corrected item-total and mean interitem correlations were > 0.4, indicating good internal consistency. Interrater reliability was assessed using ICC2,1 and kappa values. The ICC2,1 values for interrater reliability were high for all three subscales and CAT-LS grades (bedridden group: 0.974; domestic group: 0.928; community group: 0.979; and CAT-LS grade: 0.964). The kappa values for the three groups ranged from 0.898–0.927, 0.837–0.877, and 0.841–0.866, indicating substantial agreement. The Spearman’s correlation coefficients for the bedridden, domestic, and community groups were 0.852, 0.764, and 0.685, respectively, indicating good and strong concurrent validity.

**Table 9:** Distribution, internal consistency, reliability, and validity of CAT-LS.

	Bedridden group	Domestic group	Community group	CAT-LS grade
Floor/ceiling effect (%)	–	–	–	19.2/11.7
Internal consistency				
Cronbach α	0.857	0.803	0.912	0.964
Corrected-total correlation	0.724–0.750	0.619–0.721	0.809–0.852	
Mean inter-item correlation	0.657	0.463	0.694	
Inter-rater reliability (ICC_2,1_)	0.974	0.928	0.979	0.964
Kappa coefficient (κ)				
1: F1a/F2a/F3a*	0.927	0.864	0.855	
2: F1b/F2b/F3b*	0.923	0.837	0.866	
3: F1c/F2c/F3c*	0.898	0.877	0.841	
Concurrent validity^†^				
Spearman correlation coefficient	0.852	0.764	0.685	0.894

*SD* standard deviation, *ICC2,1* intraclass correlation coefficient based on two-way random effects.

^†^Compare with BI total score.

–No value.

*F1a represents the bladder and bowel, F1b represents the feeding, F1c represents the entertainment, F2a represents the toileting, F2b represents the grooming and bathing, F2c represents the housework, F3a represents the exercise in the community mobility, and F3b represents the shopping. F3c represents the social participation.

### Comparison of the question burden and time consumption between CAT-LS and BI

The CAT-LS model required fewer questions to be answered than the complete BI questionnaire, with four questions for the bedridden group and five each for the domestic and community groups. This represents a 60% and 50% decrease in question burden, respectively. The time consumption of the CAT-LS was significantly lower than that of the BI, with a median difference ranging from 9.6 to 23.7 s (Table 10).

**Table 10:** Time consumption comparison of CAT-LS and BI.

	CAT-LS (seconds)		BI (seconds)		Decrease of questions/median difference	Test	P-value
	Mean	SD	Mean	SD			
Number of questions required	4/5		10		60%/50%		
Bedridden group	25.1	18.7	37.6	23.6	12.5	*t.test*	< 0.05
Domestic group	32.5	21.4	56.2	20.5	23.7	*t.test*	< 0.05
Community group	19.6	16.3	29.2	12.1	9.6	*t.test*	< 0.05

*SD* standard deviation.

P < 0.05 indicated statistical significance.

## Discussion

Our findings demonstrate that the CAT-LS exhibits robust concurrent validity and interrater reliability. The CAT-LS encompasses only 4 out of the 5 evaluation items, reducing the assessment workload by 40% required by BI. Additionally, administering the CAT-LS takes significantly less time, with an average duration of 19.6–25.1 s, which is merely half of the time required for the BI measure. These results show the reliability, validity, and efficiency of the CAT-LS in hospitalized stroke survivors. Healthcare practitioners can confidently employ the CAT-LS to alleviate the assessment burden faced by both patients and administrators.

The CAT-LS effectively categorized 7151 patients into three groups, namely, bedridden, domestic, and community, based on their ADL levels with significantly varying BI total scores, which is similar to our previous findings using LS in neurological diseases^14^. The CAT-LS decision-tree scoring method matched stroke survivors’ levels of ADL. A large proportion (56.2%) of stroke survivors were classified as bedridden groups, which can be attributed to these participants being hospitalized with poor ADL scores compared to community stroke survivors.

Significant differences were observed in the median scores for each BI item across the three CAT-LS groups. Specifically, the bedridden group exhibited lower scores in several items compared to the domestic group, including bathing, grooming, dressing, toileting, bed/chair transfer, walking, and climbing stairs. Notably, in the bedridden group, BI item scores for bathing and climbing stairs were consistently low at approximately 0 points. In contrast, the community group consistently achieved the highest scores, particularly in domains such as feeding, grooming, dressing, bowel management, bladder management, toileting, and bed/chair transfers with perfect scores of 10 or 15. These findings suggest that without directly querying patients about their ability to perform tasks such as bathing, walking, climbing stairs, or using the toilet, we can reasonably infer that stroke survivors in the bedridden group would likely require the most assistance in these specific activities. Conversely, stroke survivors in the community group did not feel necessary to assess their ability level concerning feeding, bladder management, and bowel management indicating self-sufficiency in these areas without external assistance. In the CAT-LS evaluation results, the score difference of each BI item among the three CAT-LS groups precisely shows that it is unnecessary to evaluate all BI items, as the approximate level of help of each BI item of the evaluation object can be known.

The results of the CAT-LS decision-tree scoring model were based on the individual transfer ability and mobility scope, and classified individuals into three groups. Once the participants were classified into one of the three groups, only the items in that group were evaluated. A decision tree is a versatile predictive model that learns based on observations and logic^34^. It represents and classifies events using a rule-based forecasting system^34^. The CAT algorithm selected the most appropriate question to be asked next using information from questions already answered^18,35^, and each subscale item was evaluated based on group classification.

Our results indicate that the CAT-LS demonstrates acceptable psychometric properties for evaluating ADL in stroke survivors. Concurrent validity was assessed by calculating Spearman’s correlation coefficients between the CAT-LS and BI, a commonly used ADL assessment tool^27^. The correlation between CAT-LS grades and total BI score was 0.964 in hospitalized stroke survivors, indicating good concurrent validity, similar to that of LS used in other diseases^11,26^. Here, the large dataset used is a critical advantage that supports the statistical analysis performed. The linear regression analysis of the CAT-LS results and BI scores showed an ideal correlation coefficient for standard validity (R^2^ = 0.874). The high R^2^ values indicated that the CAT-LS results were closely associated with the model’s predictions of the total BI score in stroke survivors. These findings indicate that the scores of the CAT-LS are likely to be strongly correlated with those of the BI. If so, the scores of the CAT-LS items about BADL and those of the BI are comparable and even interchangeable using linear transformation.

The CAT-LS grades had floor and ceiling effects of < 20%, indicating that the tool was sensitive to changes in ADL ability across the full range of scores. The CAT-LS grades demonstrated floor and ceiling effects of 19.2/11.7, which fell below the recommended level of 20%, as suggested in previous studies^36,37^. The internal consistency of three CAT-LS groups, as measured using Cronbach’s α, was 0.857, 0.803, and 0.912, respectively. Cronbach’s α between 0.70 and 0.95 indicates good internal consistency^38^. Therefore, the internal consistency of all three groups was deemed acceptable and comparable to that of the traditional version of the LS^11^. Interrater reliability was high, as evidenced by the ICC2,1 values ranging from 0.928 to 0.979, as well as the kappa values falling within the range of 0.898–0.927, 0.837–0.877, and 0.841–0.866 for the bedridden, domestic, and community groups, respectively. The above results suggest that the CAT-LS showed good consistency with the BI in classifying the ADL groups and high interrater reliability when used in stroke survivors.

With approximately 2.5 million new stroke cases yearly, the number of patients requiring ADL assessment and assistance is expected to increase exponentially in China^39^. The inner logic of the programming makes the CAT-LS evaluation process easy to implement and reduces the administrative burden on both clinicians and patients. The decision tree used in CAT-LS reduces the number of questions required by 50% or 60% compared with BI, promoting measurement efficiency^40^. CAT-LS can streamline the ADL assessment process and alleviate the burden on healthcare professionals. Therefore, CAT-LS has great potential for use by clinicians and patients in time-pressed clinical settings to effectively manage stroke survivors.

### Study limitations

Although our findings are positive, this study had some limitations. First, this was a cross-sectional cohort study; we did not test the response validity in a clinical setting. Therefore, the sensitivity of the CAT-LS to changes over time should be further explored; and it could be combined with a longitudinal study to gain insight into the responsiveness of the CAT-LS to changes in ADLs over time. Second, the questionnaire is currently limited to the Chinese population, which may limit the generalisability of the study. However, we already planned to validate the validity of the CAT-LS in multiple languages, carrying out validation in different populations, languages, and settings to ensure its applicability. Third, the stroke survivors’ data were from hospitals; participants in the acute stage of stroke are unlikely to perform IADL (e.g., washing clothes, housework, or shopping), which could have introduced information bias. Therefore, the generalizability of our results may be limited. In terms of methodological constraints, using structural equation modeling (SEM), particularly the non-parametric approach Partial Least Squares (PLS)^41^, is indeed a suitable method for analyzing correlations between latent variables such as CAT-LS and BI. This is especially relevant when the sample is not a random list of patients. PLS-SEM is well-suited for small sample sizes, non-normal data, and complex relationships between variables.

## Conclusions

The CAT-LS demonstrated strong performance as a tool for evaluating the ADL of stroke survivors, with high concurrent validity and interrater reliability. Notably, the CAT-LS using the tree-decision method can alleviate the assessment burden on patients and examiners by reducing the number of items. The assessment results could be incorporated into electronic medical records to further improve efficiency in patient management in the clinic setting.

## Data Availability

All the summarized and analysed data during this study are included in this published article; the original data in this study are available from the corresponding author upon reasonable request.

## Abbreviations

ADL: Activities of daily living
BADL: Basic activities of daily living
BI: Barthel index
CAT-LS: Computer-adaptive test-Longshi scale
CAT: Computerized adaptive testing
IADL: Instrumental activities of daily living
ICC2,1: Intraclass correlation coefficient based on a two-way random effect
ICF: International Classification of Functioning, Disability, and Health
LS: Longshi scale
QRL: Quicker recovery line
